# A novel SNP assay reveals increased genetic variability and abundance following translocations to a remnant Allegheny woodrat population

**DOI:** 10.1186/s12862-022-02083-w

**Published:** 2022-11-24

**Authors:** Megan Muller-Girard, Gretchen Fowles, Joseph Duchamp, Samantha Kouneski, Cheryl Mollohan, Timothy J. Smyser, Gregory G. Turner, Bradford Westrich, Jacqueline M. Doyle

**Affiliations:** 1 Department of Environmental Science and Studies, Towson University, 8000 York Rd, Baltimore, MD 21252 USA; 2 Endangered and Nongame Species Program, New Jersey DEP Fish and Wildlife, 1255 County Rd 629, Lebanon, NJ 08833 USA; 3 Department of Biology, Indiana University of Pennsylvania, 975 Oakland Avenue, Indiana, PA 15705-1081 USA; 4 Department of Biological Sciences, Towson University, 8000 York Rd, Baltimore, MD 21252 USA; 5 WildWork, 16125 West Prosperi Avenue, Tucson, AZ 85736 USA; 6 USDA-APHIS-WS National Wildlife Research Center, Fort Collins, CO USA; 7 Pennsylvania Game Commission, 2001 Elmerton Avenue, Harrisburg, PA 17110 USA; 8 Indiana Department of Natural Resources, 5596 East State Road 46, Bloomington, IN 47401 USA

**Keywords:** Reproductive skew, Inbreeding, Genetic restoration, Inbreeding depression, Translocation, Landscape genetics, Dispersal

## Abstract

**Background:**

Allegheny woodrats (*Neotoma magister*) are found in metapopulations distributed throughout the Interior Highlands and Appalachia. Historically these metapopulations persisted as relatively fluid networks, enabling gene flow between subpopulations and recolonization of formerly extirpated regions. However, over the past 45 years, the abundance of Allegheny woodrats has declined throughout the species’ range due to a combination of habitat destruction, declining hard mast availability, and roundworm parasitism. In an effort to initiate genetic rescue of a small, genetically depauperate subpopulation in New Jersey, woodrats were translocated from a genetically robust population in Pennsylvania (PA) in 2015, 2016 and 2017. Herein, we assess the efficacy of these translocations to restore genetic diversity within the recipient population.

**Results:**

We designed a novel 134 single nucleotide polymorphism panel, which was used to genotype the six woodrats translocated from PA and 82 individuals from the NJ population captured before and after the translocation events. These data indicated that a minimum of two translocated individuals successfully produced at least 13 offspring, who reproduced as well. Further, population-wide observed heterozygosity rose substantially following the first set of translocations, reached levels comparable to that of populations in Indiana and Ohio, and remained elevated over the subsequent years. Abundance also increased during the monitoring period, suggesting Pennsylvania translocations initiated genetic rescue of the New Jersey population.

**Conclusions:**

Our results indicate, encouragingly, that very small numbers of translocated individuals can successfully restore the genetic diversity of a threatened population. Our work also highlights the challenges of managing very small populations, such as when translocated individuals have greater reproductive success relative to residents. Finally, we note that ongoing work with Allegheny woodrats may broadly shape our understanding of genetic rescue within metapopulations and across heterogeneous landscapes.

**Supplementary Information:**

The online version contains supplementary material available at 10.1186/s12862-022-02083-w.

## Background

Landscape-scale anthropogenic disturbance can cause habitat loss and fragmentation, thereby spatially isolating local wildlife populations and impeding functional connectivity [1–4]. Species that typically structure as metapopulations may be particularly threatened by spatial isolation of subpopulations. Local extirpations at patches can be common, and persistence of the metapopulation is dependent on ongoing recolonization events [5–7]. As such, interrupted dispersal and gene flow among habitat sites can decrease population-wide genetic variability and fitness, promote extirpation of naturally small subpopulations, prevent recolonization events and threaten metapopulation persistence [4, 5, 8, 9]. Allegheny woodrats (*Neotoma magister*) require rock habitats (e.g., cliff faces, talus slopes, boulder fields), located primarily in high elevation areas throughout the Appalachian Mountains and Interior Highlands [10, 11]. These habitat sites are naturally disjunct. As a result, woodrats typically form small subpopulations (defined by suitable rock habitat) that are connected by dispersal within a larger metapopulation [12, 13]. If movement amongst habitat patches is interrupted, subpopulations become isolated, gene flow is inhibited, genetic diversity is lost through drift, inbreeding depression occurs, population numbers decrease, and recolonization of extirpated sites declines [14–16].

New Jersey (NJ) is home to a single remnant population of Allegheny woodrats, located ~ 240 km from the nearest extant population in Pennsylvania. Individuals sampled in 2009, 2011, and 2012 and genotyped at 11 microsatellite loci had relatively low genetic variability, as indicated by allelic diversity and observed heterozygosity (Additional file 1). In response to conservation concerns associated with declining genetic diversity, New Jersey DEP Fish and Wildlife introduced six individuals from a genetically robust population in Pennsylvania in 2015, 2016, and 2017 under the assumption that, if translocated individuals reproduced, population numbers and genetic variability would increase (i.e., genetic rescue; [17–20]). However, identifying evidence of reproductive success and quantifying genetic variability are dependent on identifying a marker panel with suitable statistical power [21–23].

Panels of single-nucleotide polymorphisms (SNPs) can yield a low probability of identity (P_ID_) (i.e., the likelihood that two randomly chosen individuals in a population will present seemingly identical genotypes; [21]), thus aiding accurate reconstruction of familial relationships [24–26], even when populations are inbred [23]. Even relatively small SNP panels (e.g., 58–109 markers) can ultimately perform as well or better than small suites of microsatellites [23, 27–30]. To this end, we sequenced the Allegheny woodrat genome and annotated a draft genome assembly. We subsequently designed a 134 SNP panel incorporating both gene-associated and putatively neutral markers. We conducted preliminary analyses to explore whether the SNP assay provides greater statistical power for individual identification than a commonly used panel of microsatellite markers. The SNP loci were then used to evaluate changes in genetic diversity following translocations to New Jersey’s remnant population and identify offspring of translocated individuals.

## Results

### Nuclear genome sequencing and SNPtype assay development

We generated 137.6 gigabases (Gb) of raw sequence data from *N. magister*, including 119.8 Gb from the paired-end (PE) library and 17.8 Gb from the mate-paired (MP) library (Additional File 2). Our draft nuclear genome assembly includes 60,789 scaffolds greater than 2000 basepairs (bp) in length. We used BUSCO v5 to evaluate completeness of the genome by identifying mammalia_odb10 orthologs, finding 77.9% of universal single-copy orthologs were complete (77.2% single copy, 0.7% duplicated), 8.7% fragmented and 13.4% missing.

We initially identified 627,421 high-quality SNPs. Of these, we selected 192 SNPs to include in a Fluidigm SNPtype assay. We subsequently excluded 58 loci for reasons outlined in the methods (e.g., data did not cluster into distinct homozygous and heterozygous states). Of the remaining 134 loci, at least 128 loci amplified for each of the 318 woodrats genotyped (Additional File 3). These loci were roughly divided between gene-associated (n = 72) and neutral markers (n = 62).

### Probability of identity using microsatellite and SNP markers

Genotyping 50 woodrats captured in 2017 and 2019 in Adams County, Ohio (OH) at 11 microsatellite markers resulted in a P_ID_ of 4.0 × 10^–5^ and a probability of identity among siblings (PIDsib) of 9.8 × 10^–3^. By contrast, 134 SNP loci generate values of 5.0 × 10^–27^ (P_ID_) and 3.1 × 10^–14^ (PIDsib). If the more conservative data set of 70 loci is used, the P_ID_ is 1.9 × 10^–13^ and the PIDsib is 3.1 × 10^–7^ across all 50 individuals. Furthermore, our results indicate that a much smaller panel of SNPs might be utilized in subsequent studies to achieve a P_ID_ < 0.0001 (Additional File 4; a PID < 0.0001 is considered low enough to distinguish between even closely related individuals in most natural populations [31–33]). Given these results, all other samples were genotyped using just the SNP panel. Notably, there was a significant, positive relationship between the number of heterozygous microsatellite loci per individual and the number of heterozygous SNP loci per individual (linear regression: r^2^ = 0.32, p < 0.0001, Additional File 5).

### Genetic variability and reproductive success following translocations to the Palisades population

Parentage analysis revealed that the six woodrats translocated from Pennsylvania to New Jersey produced a minimum of thirteen offspring (Table 1). The female translocated in 2015 produced at least four offspring between 2016 and 2019. The male translocated in 2015 produced at least nine offspring, predominantly in 2016 (Table 1). The offspring of the 2015 translocated female and male produced at least thirteen offspring of their own between 2017 and 2019 (Table 1). We found no evidence from trapping and subsequent genotyping that the other four translocated individuals reproduced. The males translocated in 2016 and 2017 were confirmed dead within 11 and 1 weeks of release, respectively. A camera captured footage of the female translocated in 2016 with a pup. It is unclear whether this pup died before reaching adulthood or simply avoided trapping, as the location in which it was photographed was outside of the regular trapping area. The female translocated in 2017 also settled outside of the regular trapping area and was not detected again.

**Table 1:** Offspring of individuals translocated from Pennsylvania in 2015, 2016 and 2017 and their offspring

ID	Year translocated/first captured	Sex	Offspring identified with paternity analysis			
			2016	2017	2018	2019
F001^a^	2015	Female	F028, F029, M030		M135	
M002	2015	Male	F014, M015, F019, M020, F022, F023, F025	F100	F138	
M012	2016	Male				
F013	2016	Female				
M128	2017	Male				
F129	2017	Female				
F019^b^	2016	Female		F265	F138	
M020	2016	Male		F090		F245
F029	2016	Female		M102	F134	
M030	2016	Male			F134, M136	
F090	2017	Female			M131, M132	
M102	2017	Male				M261
F133	2018	Female				F250
M135	2018	Male				M252

^a^Individuals F001, M002, M012, F013, M128 and F129 were translocated from Pennsylvania to New Jersey in 2015, 2016 and 2017

^b^Individuals F019, M020, F029, M030, F090, M102, F133, and M135 are the 2^nd^ and 3^rd^ generation progeny of translocated individuals for which there is evidence of reproduction

**Fig. 1:**
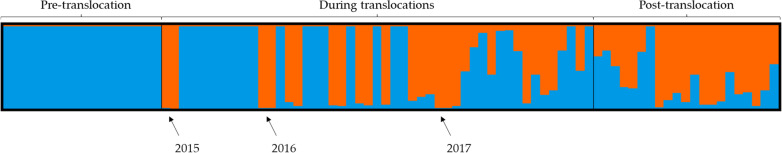
STRUCTURE results for 82 woodrats trapped in the Palisades, NJ between 2009 and 2019, as well as six individuals translocated from PA to NJ. PA individuals are labeled with the years in which they were released in NJ (i.e., 2015, 2016 and 2017). All individuals are labeled as being sampled before translocations occurred (“pre-translocation”), during translocations or after translocations occurred (“post-translocation”). STRUCTURE results were CLUMPP-averaged across 10 runs when K is assumed to be equal to two. Admixture is indicated by a shift from the blue cluster associated with the resident population prior to human-mediated gene flow, to an increase in the orange cluster associated with the genetic profiles of the PA individuals

Of the 82 tissue samples collected from the Palisades population, five were collected in 2009, thirteen in 2011, nine in 2015, eighteen in 2016, sixteen in 2017, eight in 2018 and thirteen in 2019. Once a Bonferroni correction was applied, a single locus was found to be out of Hardy–Weinberg equilibrium, and only in 2019 (exhibiting evidence of heterozygote excess). STRUCTURE analysis of resident individuals and those translocated to NJ provided evidence of two genetically distinct clusters when the most conservative data set (70 loci) was utilized. The population-wide genetic composition changed following translocation events in 2015, as illustrated by a shift from the blue cluster associated with the resident population prior to human-mediated gene flow, to an increase in the orange cluster associated with the genetic profiles of the PA individuals (Fig. 1). Despite this, all alleles historically present at the loci considered in this study were retained following translocations (data not shown).

**Fig. 2:**
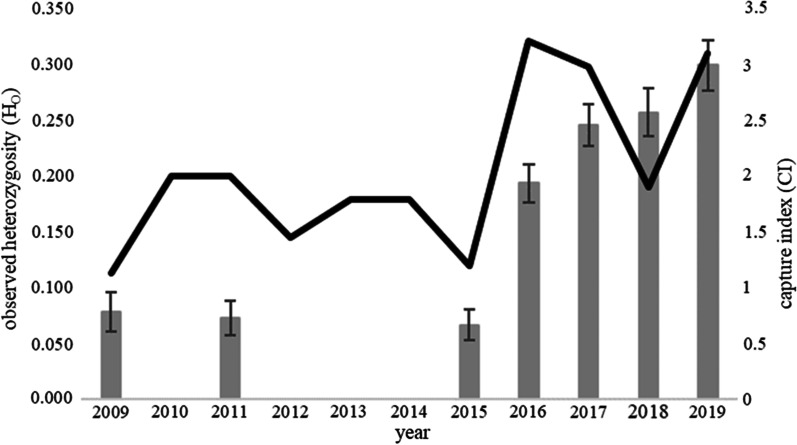
Capture index and mean observed heterozygosity (H_O_) ± SE for Allegheny woodrats (*Neotoma magister*) captured in 2009 (n = 5), 2011 (n = 13), 2015 (n = 9), 2016 (n = 18), 2017 (n = 16), 2018 (n = 8) and 2019 (n = 13) in the Palisades, NJ and genotyped at 134 SNP loci. Observed heterozygosity increased following translocations of six woodrats from Pennsylvania in 2015, 2016 and 2017

Prior to translocations, observed heterozygosity (H_O_) and expected heterozygosity (H_E_) were substantially lower in New Jersey than at sites in Indiana (IN) and Ohio (Table 2). However, genetic variability in the New Jersey population increased notably in the years following translocation (Table 2, Table 3, Fig. 2). For example, observed heterozygosity increased from 0.08 ± 0.02 in 2009 to 0.30 ± 0.02 in 2019 (Table 3, Fig. 2). Average H_O_ and H_E_ were comparable in Indiana, Ohio and (post-translocation) New Jersey (Tables 2, 3, Fig. 2).

**Table 2:** Mean observed heterozygosity (H_O_) ± SE, mean expected heterozygosity (H_E_) ± SE for Allegheny woodrats (*Neotoma magister*) genotyped at 134 SNPs

Year	Females	Males	H_O_ ± SE	H_E_ ± SE
Indiana	82	90	0.27 ± 0.01	0.23 ± 0.01
Ohio	31	27	0.25 ± 0.02	0.23 ± 0.02
Pennsylvania^a^	3	3		
New Jersey—pre-translocation^b^	9	9	0.08 ± 0.02	0.07 ± 0.01
New Jersey—during translocations	23	20	0.21 ± 0.02	0.18 ± 0.01
New Jersey—post-translocation	10	11	0.27 ± 0.02	0.24 ± 0.02

^a^Observed and expected heterozygosity were not calculated for the 6 individuals from Pennsylvania

^b^Summary statistics for individuals captured before (2009, 2011), during years in which translocations also occurred (2015–2017) and after (2018–2019) translocations occurred are indicated by “pre-translocation”, “during translocations” and “post-translocation”, respectively

**Table 3:** Mean observed heterozygosity (H_O_) ± SE, mean expected heterozygosity (H_E_) ± SE and mean number of alleles (A) for Allegheny woodrats (*Neotoma magister*) captured in 2009 (n = 5), 2011 (n = 13), 2015 (n = 9), 2016 (n = 18), 2017 (n = 16), 2018 (n = 8) and 2019 (n = 13) in the Palisades, NJ and genotyped at 134 SNP loci

Year	H_O_ ± SE	H_E_ ± SE	A
2009	0.08 ± 0.02	0.06 ± 0.01	1.2
2011	0.07 ± 0.02	0.07 ± 0.01	1.2
2015	0.07 ± 0.01	0.07 ± 0.01	1.2
2016	0.19 ± 0.02	0.17 ± 0.01	1.6
2017	0.25 ± 0.02	0.21 ± 0.02	1.7
2018	0.26 ± 0.02	0.21 ± 0.02	1.7
2019	0.30 ± 0.02	0.25 ± 0.02	1.7

We identified 20 publications for which a Fluidigm SNPtype assay was used to genotype individuals at relatively few loci (38–192 SNPs) and H_O_ and/or H_E_ were reported (Table 4). Species described were members of Actinopterygii, Aves, Bivalvia, and Mammalia and predominantly considered of “Least concern” by the IUCN. Across studies, H_O_ and H_E_ ranged from 0.13 to 0.45 and 0.14 to 0.42; respectively (Table 4). The vast majority of H_O_ and H_E_ estimates for species characterized as “Least concern” (all but two) fell between 0.25 and 0.37. Median H_O_ and H_E_ were 0.32 and 0.31, respectively.

**Table 4:** Metrics of genetic variability, sample size and IUCN status for species genotyped using the Fluidigm® BioMark, HD™ Genotyping System

Class	Species	IUCN status	Sample size	Number of loci	Ho	He	Citation
Actinopterygii	*Tripterygion delaisi*	LC	1599	192	0.45	N/A	[94]
	*Alosa pseudoharengus*	LC	323	96	0.25	0.25	[95]
			5678	92	0.25	0.25	[96]
	*Alosa aestivalis*	VU	433	95	0.29	0.29	[95]
			2247	95	0.29	0.29	[96]
	*Oncorhynchus tshawytscha*	N/A	188	91	0.34	0.34	[97]
			8031	96	0.32	N/A	[97]
	*Oncorhynchus mykiss*	N/A	6165	96	0.41	0.42	[98]
Aves	*Aquila chrysaetos*	LC	52	159	0.33	0.34	[24]
			344	159	0.34	0.34	[99]
	*Cyrtonyx montezumae*	LC	186	169	0.32	0.35	[100]
	*Falco mexicanus*	LC	103	143	0.34	0.34	[26]
Bivalvia	*Crassostrea virginica*	N/A	560	38	0.28	N/A	[101, 102]
Mammalia	*Ovis dalli dalli*	LC	476	188	0.29	0.31	[42]
	*Macaca fascicularis*	VU	129	83	0.13	0.14	[103]
	*Canis lupus*	LC	40	85	0.31	0.29	[32]
	*Felis silvestris*	LC	37	65	0.16	0.35	[32]
				96	0.37	0.45	[33]
	*Ursus arctos*	LC	70	69	0.28	0.31	[32]
	*Eschrichtius robustus*	LC	28	88	0.32	0.31	[25]

## Discussion

### Genome sequencing and SNP assay development

The data described herein represent only the second time a member of the genus *Neotoma* has undergone whole-genome sequencing [34]. Genetic resources for *Neotoma magister* are particularly limited [35], yet even low coverage sequencing can be used to generate tools that inform management of threatened species. For example, two lanes of paired-end sequencing and one lane of mate-paired sequencing enabled assembly of the complete mitochondrial genome [35] and identification of the 134 SNP loci described in this manuscript. Studies have shown that in some cases SNP genotyping can better reveal fine-scale population structure, provide evidence of differential selection amongst populations and estimate genome wide heterozygosity than other marker panels [36–44]. Genotyping 50 woodrats using both microsatellite and SNP loci indicates that our SNP assay provides increased statistical power for analyses. Furthermore, PIDsib estimates are also very low, indicating the panel can be used to distinguish between woodrats even when related individuals are present in the population [31–33]. Given the spatial isolation of many woodrat populations (e.g., the remnant NJ Palisades population), the presence of closely related individuals should be assumed. Finally, DNA extracted from naturally shed feathers, hair and fecal samples and subsequent Fluidigm SNP genotyping has been used to identify individual golden eagles [24], wolves, wildcats, and bears [32, 33]. Given the low P_ID_ estimates associated with this assay, we anticipate a similar approach could be used to non-invasively monitor Allegheny woodrat populations from hair or fecal samples.

### Temporal shifts in genetic variability following translocations to New Jersey’s remnant population

Conservation managers have long worried that translocations across extended geographic distances would result in relatively greater genetic distance amongst introduced and resident individuals, increasing the risk of outbreeding depression [45]. Recent studies, however, indicate that outbreeding depression rarely has negative impacts on the success of translocation programs [19, 46]. Furthermore, factors such as the genetic diversity of translocated individuals may be better predictors of fitness following introduction to a novel population than genetic distance [47]. Despite the relatively great geographic distance between source and resident populations inherent in this study, successful reproduction by translocated individuals clearly drove increases in genetic variability in subsequent years. Parentage analysis provides evidence that at least two woodrats translocated from Pennsylvania to New Jersey in 2015 went on to reproduce, as did their offspring. Increases in H_O_ and H_E_ were apparent as soon as 2016, making observed levels comparable to those among woodrat populations in IN and OH, and persisted through the end of the monitoring period in 2019. We also compared genetic variability in the NJ population to that of other species and determined that observed heterozygosity of New Jersey’s woodrats caught before 2015 was notably lower than any species listed as “Least concern” by the IUCN. Following translocations, H_O_ and H_E_ for the NJ population fall within the range of estimates generated across species. Increased abundance since 2015 provides additional evidence of potential genetic rescue. As such, this study joins relatively few in providing evidence of an increase in population size or growth rate following assisted gene flow (reviewed in [19]).

### Management implications

Ongoing research on the conservation of Allegheny woodrats may inform best practices in translocating individuals to very small populations. Management guidelines recommend translocating a number of non-resident individuals that represent 20% of the recipient population to minimize the likelihood of swamping out local adaptive genetic variation [17, 20]. Genetic swamping (i.e., the rapid increase in frequency of alleles introduced by gene flow; [48, 49]) can result in the loss of private alleles within the recipient population. This, in turn, can lead to a loss of species-wide allelic diversity [49], even as the resident population’s genetic diversity increases. Efforts to minimize genetic swamping can lead to translocating very few individuals when recipient populations have low abundance. This study joins others in suggesting that successful reproduction by just one to three non-resident individuals can promote increased genetic diversity and abundance [50–53]. In some cases, however, these few immigrants achieve substantially elevated reproductive success in comparison to resident individuals, contributing to inbreeding in subsequent generations (e.g., arctic foxes, [51]; wolves, [54–56]). Even in the absence of direct observations of inbreeding, reproductive skew is known to decrease effective population size and result in the accelerated loss of genetic diversity due to drift [57]. Disproportionate reproductive success by translocated individuals may prove to be common in Allegheny woodrats if sex ratios are skewed in small populations [50], resident individuals prefer to mate with translocated individuals as an inbreeding avoidance mechanism [58], or F1 offspring have increased fitness stemming from heterosis [59–61]. Both this study and Davis et al., [50] document observations of translocated male Allegheny woodrats siring 39 and 35% of young trapped in the subsequent season, in their respective populations. A few known instances of inbreeding amongst relatives followed in subsequent generations (Table 2, [50]) but, encouragingly, coincided with stable or increasing population-wide genetic variability and abundance. Conservation managers monitoring small populations might consider genotyping individuals, conducting parentage analyses, and monitoring genetic diversity on a bi-yearly or yearly basis. This would allow for rapid translocation of additional individuals to small populations if elevated reproductive success seems likely to lead to inbreeding events, or, to supplement previous, unsuccessful attempts at genetic rescue (i.e., if non-resident survivorship is low). It is worth noting that even in the absence of inbreeding, truly isolated populations (like that of the Palisades) will ultimately require additional human-mediated gene flow to counteract loss of genetic diversity due to genetic drift.

There are additional ways in which ongoing studies of woodrat translocations have the potential to add depth to our understanding of genetic rescue and restoration. Studies of genetic rescue have typically considered populations as discrete units, uninterrupted by landscape features. The natural tendency for woodrats to exist in metapopulations give scientists the opportunity to study genetic rescue throughout heterogeneous landscapes and, in particular, how alleles introduced at one habitat patch have the potential to move amongst sites. Just as recent studies have proposed choosing specific individuals for their ability to reduce inbreeding depression in genetically depauperate populations [19, 62], specific habitat sites might be targeted for releases if they are connected by natural dispersal corridors to other portions of the metapopulation. Indeed, recent work on the landscape genetics of Virginia’s Allegheny woodrats suggests that low elevation, rather than anthropogenic barriers such as roads, might prevent translocated individuals and/or their offspring from dispersing amongst habitat sites [63].

## Conclusions

Herein, we describe a novel SNP assay, which provides increased statistical power to studies of a species commonly found in small and consanguineous populations. Our study has important implications for remnant populations of threatened species that are geographically isolated from the nearest metapopulation. Translocating small numbers of individuals to very small populations may increase the risk of reproductive skew followed by genetic drift and inbreeding, necessitating increased monitoring following introductions. Despite this, human-mediated gene flow is likely to be integral to the persistence of remnant populations. Our results indicate, encouragingly, that small numbers of introduced, genetically variable individuals can successfully reproduce, increase population-wide genetic diversity, and facilitate increased abundance.

## Methods

### Genome assembly and annotation

We extracted deoxyribonucleic acid (DNA) from a tail clip of a single *N. magister* individual by pairing commercially available extraction (DNEasy Blood and Tissue, Qiagen, Venlo, the Netherlands) and clean-up (DNA clean & Concentrator, Zymo Research, Irvine, California) kits in accord with the manufacturers’ instructions. We conducted three lanes of paired-end and one lane of mate-paired sequencing using an Illumina HiSeq2500. We used Trimmomatic [64] to remove adaptors, discard short reads and trim poor quality bases from 5′ and 3′ ends of raw sequence reads as described in Schofield et al., [35]. We used ABySS 1.9.0 [65] to conduct several assemblies with kmer lengths ranging from 40 to 85. PE reads were used to generate contigs. MP reads were used to infer the order, orientation, and distance between contigs, linking them together in scaffolds. The assembly with the greatest N50 value and longest scaffold was used for downstream analyses. BUSCO v5 [66], implemented by gVolante [67], was used to evaluate completeness of the genome.

We used the MAKER 2.28 pipeline [68] to annotate all scaffolds greater than 10 kb, following the methods described in Doyle et al. [69] and Doyle et al. [26]. To briefly summarize, we first used Repeat-Masker to identify and mask stretches of repetitive DNA. Second, we downloaded 6762 *Mus musculus* protein sequences from the UniProtKB database (www.uniprot.org) and used the protein2genome setting in MAKER to generate gene annotations. These annotations were subsequently used to train SNAP [70] and generate ab initio predictions. Third, we aligned protein sequences and 93,400 *Mus musculus* expressed sequence tag (EST) sequences to the genome using BLAST and used InterProScan to identify putative protein domains. Finally, all ab initio gene predictions supported by protein, EST or InterProScan evidence were promoted to gene annotations.

### SNP discovery and assay design

We identified SNPs as in Doyle et al. [26]. Briefly, we aligned paired-end reads back to the draft genome assembly using BWA 0.7.12 [71] and used Picard 2.3 (http://broadinstitute.github.io/picard) to sort and identify duplicate reads. We used the GATK 3.6 pipeline [72, 73] to realign reads around indels and identify high quality SNPs with a Phred quality score ≥ 30. We then selected 95 autosomal nuclear markers associated with gene deserts (i.e., “neutral” markers) and 97 autosomal nuclear markers associated with protein-coding genes. We deliberately chose no more than one SNP of each category from a given scaffold to minimize linkage disequilibrium. To identify neutral markers, we quantified the distances between all SNPs and genes using the BEDtools suite [74], ultimately choosing markers at the 95% percentile distance from genes. We used SnpEff 4.3 [75] to find SNPs associated with non-synonymous changes in the exonic regions of genes (i.e., “gene-associated” markers). IGV 2.3 [76] was used to confirm that at least 60 nucleotides of high-quality flanking sequence were present upstream and downstream of the marker, that guanine-cytosine (GC) content was less than 65%, and that no other variable sites were present within 20 nucleotides.

### DNA extraction and SNP genotyping

We trapped and subsequently genotyped 82 woodrats sampled from the Palisades, NJ between 2009 and 2019 (Tables 2, 3), including 18 and 64 individuals sampled before and after translocations began, respectively. We followed standard live-trapping protocols [77, 78] and collected a 2-mm ear punch from each individual, which was preserved in 70–100% ethanol. For each year trapping occurred, we calculated a capture index by dividing the number of unique individuals caught by the number of trap nights and multiplying by 10 [79]. Calculating a trap index allows us to control for differences in the number of nights trapping occurred across years [77]. DNA extractions were performed using an ammonium acetate protocol [14] or the Zymo Quick-DNA Miniprep Plus Kit. We used the Fluidigm® BioMark HD™ Genotyping System to genotype these individuals. Additionally, we genotyped the six individuals translocated from Pennsylvania to New Jersey between 2015 and 2017. Finally, we opportunistically genotyped 172 and 58 samples collected from Indiana and Ohio, respectively (Table 2). These samples were collected between 2015 and 2019 as part of long-term monitoring studies.

SNP calls were edited using the Fluidigm® Genotyping Analysis Software. We excluded markers from downstream analysis when data did not cluster into distinct homozygous and heterozygous states and if minor allele frequencies were less than 0.025. We used chi-squared tests implemented by GenAlEx [24, 26] to test for departures from Hardy–Weinberg equilibrium. Following Bonferroni correction, a single locus was found to be consistently out of Hardy–Weinberg equilibrium across years and was omitted, leaving 134 loci. We excluded individuals from analysis if ≥ 7 loci were not successfully genotyped, as call rates tend to be negatively correlated with genotyping errors [24, 32]. Using snpStats [80], we identified a number of markers in linkage disequilibrium but assumed that in many cases this was due to consanguinity, rather than two markers being in close proximity along the genome. However, to meet the assumptions of Cervus 3.0.7 [81] and STRUCTURE 2.3.4 [82, 83], we identified all pair-wise comparisons with r^2^ > 0.2 and removed one marker in each case, creating a reduced dataset of 70 loci in which all SNPs are in linkage equilibrium.

### Probability of identity using microsatellite and SNP markers

Previous studies of *N. magister* utilized relatively small panels of 11–22 microsatellite markers (e.g., [15, 63, 84, 85]). To evaluate the statistical power associated with each approach, we genotyped 50 woodrats captured in 2017 and 2019 in the Adams County, OH at both 11 microsatellites and 134 SNP loci (Additional File 3 and Additional File 4). We subsequently calculated the probability that two randomly chosen individuals in the population would have identical genotypes (P_ID_), using each marker panel. We additionally calculated PIDsib, which represents a conservative upper bound for the likelihood that two individuals sampled from a population will have the same genotype by chance [32, 33]. This estimate is particularly useful when substructure is present in the population (i.e., related individuals; [32, 33]).

### Reproductive success and genetic variability following translocations to the Palisades

We used Cervus 3.0.7 [81] to assign individuals sampled between 2015 and 2019 to dams and sires. For individuals trapped for the first time in each year, all woodrats trapped in that same year and in all previous years were considered candidate parents. Simulations included 100,000 replicate cycles. The proportion of candidate dams and sires sampled was estimated to be 0.80, based on the probability of capture estimated from comparable trapping approaches of other woodrat populations [16]. The proportion of typed loci was 0.99 and the proportion of loci mistyped was set to 0.04 [26]. The minimum confidence level for parentage assignment was 95%.

STRUCTURE 2.3.4 [82, 83], STRUCTURE HARVESTER 0.6.94 [86], and Clumpak [87] were used to visualize admixture in the Palisades, NJ woodrat population across time. We utilized the reduced dataset (i.e., with all 70 loci in linkage equilibrium, see above) for 82 individuals sampled between 2009 and 2019, as well as the six individuals translocated from PA. We considered values of K = 1–8, running each value 10 times with an initial burn-in of 100,000 Markov chain Monte Carlo (MCMC) iterations and 1,000,000 subsequent iterations for each value. We assumed an admixture ancestry model and allowed for correlated allele frequencies [82]. The results were interpreted using mean likelihood values of K and ΔK [86].

We used GenAlEx [88] to calculate allele frequencies and expected and observed heterozygosity in the years before (2009, 2011), during (2015–2017) and after translocations (2018, 2019) to the Palisades, NJ population. To provide context for our interpretation of temporal changes in genetic variability in the Palisades population, we (1) used GenAlEx [88] to calculate allele frequencies and expected and observed heterozygosity in Indiana, New Jersey and Ohio and (2) surveyed the literature for estimates of observed and expected heterozygosity generated using the Fluidigm® BioMark HD™ Genotyping System and relatively small SNP assays (e.g., 96–192 loci). To conduct our literature review, we searched for the phrases “SNP type assay”, “SNPtype assay”, “Fluidigm SNP assay”, “Fluidigm SNP chip” and “Fluidigm Genotyping Analysis Software” in Google Scholar. For all studies of non-human animals for which observed and/or expected heterozygosity were described, we recorded the number of loci in the assay, sample size, metrics of genetic variability and International Union for Conservation of Nature (IUCN) status. If average H_O_ and H_E_ were not provided, we averaged across per-locus values or population-specific values when able. When interpreting these results, an important caveat is that we do not have a robust understanding of how SNP H_E_, H_O_ and allelic diversity vary with categorizations such as body size, conservation status, habitat, migratory behavior, taxonomic group and trophic class. In contrast, these relationships have been extensively studied utilizing estimates of genetic variation generated with microsatellite loci [89–93].

## Supplementary Information

**Additional file 1:** Microsatellite genotypes of woodrats sampled in 2009, 2011 and 2012 in the Palisades, NJ. The spreadsheets labeled “Raw Data” and “data GenAlEx” include the genotypes for twenty-eight individuals genotyped at 11 microsatellite loci. These individuals had relatively low genetic variability, as indicated by number of alleles (spreadsheets AFP and AGL), observed heterozygosity and expected heterozygosity (spreadsheet HFP).

**Additional file 2:** Summary statistics associated with nuclear genome sequencing, assembly and annotation.

**Additional file 3:** SNP genotypes of woodrats sampled in Indiana, New Jersey, Ohio and Pennsylvania. The spreadsheet labeled “Raw Data” includes the genotypes for 318 individuals genotyped at 134 SNP loci.

**Additional file 4:** Relationship between probability of identity (PID), probability of identity between siblings (PIDsib) and the number of genotyped SNP or microsatellite loci.

**Additional file 5:** Comparison of microsatellite- and SNP-based genetic variation.

## Data Availability

The datasets generated and/or analyzed during the current study are available in NCBI’s Short Read Archive (BioSample accession # SAMN25554335, BioProject accession # PRJNA802531), Dryad (https://doi.org/10.5061/dryad.rjdfn2zdb) and the supplementary files associated with this manuscript.

## Abbreviations

NJ: New Jersey
SNPs: Single nucleotide polymorphisms
Gb: Gigabases
bp: Basepairs
kb: Kilobases
PE: Paired-end
MP: Mate-paired
OH: Ohio
P_ID_: Probability of identity
PIDsib: Probability of identity among siblings
PA: Pennsylvania
H_O_: Observed heterozygosity
H_E_: Expected heterozygosity
IN: Indiana
DNA: Deoxyribonucleic acid
EST: Expressed sequence tag
GC: Guanine-cytosine
MCMC: Markov chain Monte Carlo iterations
IUCN: International Union for Conservation of Nature

## References

[1] Wilcox BA, Murphy DD (1985). Conservation strategy: the effects of fragmentation on extinction. Am Nat.

[2] Young A, Boyle T, Brown T (1996). The population genetic consequences of habitat fragmentation for plants. Trends Ecol Evol.

[3] Mikoláš M, Tejkal M, Kuemmerle T, Griffiths P, Svoboda M, Hlásny T (2017). Forest management impacts on capercaillie (*Tetrao urogallus*) habitat distribution and connectivity in the Carpathians. Landsc Ecol.

[4] Blanton RE, Cashner MF, Thomas MR, Brandt SL, Floyd MA (2019). Increased habitat fragmentation leads to isolation among and low genetic diversity within populations of the imperiled Kentucky Arrow Darter (*Etheostoma sagitta spilotum*). Conserv Genet.

[5] Saccheri I, Kuussaari M, Kankare M, Vikman P, Fortelius W, Hanski I (1998). Inbreeding and extinction in a butterfly metapopulation. Nature.

[6] Hanski I (1999). Metapopulation ecology.

[7] Dallas TA, Saastamoinen M, Schulz T, Ovaskainen O (2020). The relative importance of local and regional processes to metapopulation dynamics. J Anim Ecol.

[8] Couvet D (2002). Deleterious effects of restricted gene flow in fragmented populations. Conserv Biol.

[9] Nonaka E, Sirén J, Somervuo P, Ruokolainen L, Ovaskainen O, Hanski I (2019). Scaling up the effects of inbreeding depression from individuals to metapopulations. J Anim Ecol.

[10] Poole E (1940). A life history sketch of the Allegheny woodrat. J Mammal.

[11] Castleberry SB, Mengak MT, Ford WM (2006). Neotoma magister. Mamm Species.

[12] Hassinger J, Butchkoski C, Diefenbach D, Peles J, Wright J (2008). Managing surface rock communities for *Neotoma magister*. The Allegheny Woodrat: Ecology, conservation, and management of a declining species.

[13] Wood P, Peles J, Wright J (2008). Woodrat population dynamics and movement patterns. The Allegheny Woodrat: ecology, conservation, and management of a declining species.

[14] Smyser TJ, Duchamp JE, Johnson SA, Larkin JL, Rhodes OE (2012). Consequences of metapopulation collapse: comparison of genetic attributes between two Allegheny woodrat metapopulations. Conserv Genet.

[15] Smyser TJ, Page LK, College W, Eugene O (2012). Synergistic stressors and the dilemma of conservation in a multivariate world: a case study in Allegheny woodrats. Anim Conserv.

[16] Smyser TJ, Stauffer GE, Johnson SA, Hudson CM, Rhodes OE, Swihart RK (2016). Annual survival of Allegheny woodrats in a nonequilibrium metapopulation. J Mammal.

[17] Hedrick P (1995). Gene flow and genetic restoration: the Florida Panther as a case study. Conserv Biol.

[18] Frankham R (2015). Genetic rescue of small inbred populations: meta-analysis reveals large and consistent benefits of gene flow. Mol Ecol.

[19] Whiteley AR, Fitzpatrick SW, Funk WC, Tallmon DA (2015). Genetic rescue to the rescue. Trends Ecol Evol.

[20] Frankham R (2016). Genetic rescue benefits persist to at least the F3 generation, based on a meta-analysis. Biol Conserv.

[21] Paetkau D, Strobeck C (1994). Microsatellite analysis of genetic variation in black bear populations. Mol Ecol.

[22] Kalinowski S (2002). How many alleles per locus should be used to estimate genetic distances?. Heredity.

[23] Tokarska M, Marshall T, Kowalczyk R, Wójcik JM, Pertoldi C, Kristensen TN (2009). Effectiveness of microsatellite and SNP markers for parentage and identity analysis in species with low genetic diversity: The case of European bison. Heredity.

[24] Doyle JM, Katzner TE, Roemer GW, Cain JW, Millsap BA, McIntyre CL (2016). Genetic structure and viability selection in the golden eagle (*Aquila chrysaetos*), a vagile raptor with a Holarctic distribution. Conserv Genet.

[25] DeWoody J, Fernandez N, Brüniche-Olsen A, Antonides J, Doyle J, San Miguel P (2017). Characterization of the gray whale (*Eschrichtius robustus*) genome and a genotyping array based on single nucleotide polymorphisms in candidate genes. Biol Bull.

[26] Doyle J, Bell D, Bloom P, Emmons G, Katzner T, LePre L (2018). New insights into the phylogenetics and population structure of the prairie falcon (*Falco mexicanus*). BMC Genomics.

[27] Hauser L, Baird M, Hilborn R, Seeb LW, Seeb JE (2011). An empirical comparison of SNPs and microsatellites for parentage and kinship assignment in a wild sockeye salmon (*Oncorhynchus nerka*) population. Mol Ecol Resour.

[28] Buchanan JW, Woronuk GN, Marquess FL, Lang K, James ST, Deobald H (2017). Analysis of validated and population-specific single nucleotide polymorphism parentage panels in pedigreed and commercial beef cattle populations. Can J Anim Sci.

[29] Kaiser SA, Taylor SA, Chen N, Sillett TS, Bondra ER, Webster MS (2017). A comparative assessment of SNP and microsatellite markers for assigning parentage in a socially monogamous bird. Mol Ecol Resour.

[30] Thongda W, Zhao H, Zhang D, Jescovitch LN, Liu M, Guo X (2018). Development of SNP Panels as a new tool to assess the genetic diversity, population structure, and parentage analysis of the Eastern Oyster (*Crassostrea virginica*). Mar Biotechnol.

[31] Waits LP, Luikart G, Taberlet P (2001). Estimating the probability of identity among genotypes in natural populations: cautions and guidelines. Mol Ecol.

[32] von Thaden A, Cocchiararo B, Jarausch A, Jüngling H (2017). Assessing SNP genotyping of noninvasively collected wildlife samples using microfluidic arrays. Sci Rep.

[33] von Thaden A, Nowak C, Tiesmeyer A, Reiners TE, Alves PC, Lyons LA (2020). Applying genomic data in wildlife monitoring: development guidelines for genotyping degraded samples with reduced single nucleotide polymorphism panels. Mol Ecol Resour.

[34] Campbell M, Oakeson KF, Yandell M, Halpert JR, Dearing D (2016). The draft genome sequence and annotation of the desert woodrat *Neotoma lepida*. Genomics Data.

[35] Schofield M, Duchamp J, Larkin JL, Smyser TJ, Doyle JM, Schofield M (2018). Mitochondrial genome of an Allegheny Woodrat (*Neotoma magister*). Mitochondrial DNA Part B.

[36] Ruegg KC, Anderson EC, Paxton KL, Apkenas V, Lao S, Siegel RB (2014). Mapping migration in a songbird using high-resolution genetic markers. Mol Ecol.

[37] Bekkevold D, Helyar SJ, Limborg MT, Nielsen EE, Hemmer-Hansen J, Clausen LAW (2015). Gene-associated markers can assign origin in a weakly structured fish, Atlantic herring. ICES J Mar Sci.

[38] Malenfant RM, Coltman DW, Davis CS (2015). Design of a 9K illumina BeadChip for polar bears (*Ursus maritimus*) from RAD and transcriptome sequencing. Mol Ecol Resour.

[39] Ferchaud A-L, Pedersen SH, Bekkevold D, Jian J, Niu Y, Hansen MM (2014). A low-density SNP array for analyzing differential selection in freshwater and marine populations of threespine stickleback (*Gasterosteus aculeatus*). BMC Genomics.

[40] Limborg MT, Helyar SJ, De Bruyn M, Taylor MI, Nielsen EE, Ogden R (2012). Environmental selection on transcriptome-derived SNPs in a high gene flow marine fish, the Atlantic herring (*Clupea harengus*). Mol Ecol.

[41] DeWoody YD, DeWoody JA (2005). On the estimation of genome-wide heterozygosity using molecular markers. J Hered.

[42] Roffler GH, Amish SJ, Smith S, Cosart TED, Kardos M (2016). SNP discovery in candidate adaptive genes using exon capture in a free-ranging alpine ungulate. Mol Ecol Resour.

[43] Hoffman JI, Simpson F, David P, Rijks JM, Kuiken T, Thorne MAS (2014). High-throughput sequencing reveals inbreeding depression in a natural population. Proc Natl Acad Sci USA.

[44] Clemento AJ, Crandall ED, Garza JC, Anderson EC, Garza JC, Anderson EC (2014). Evaluation of a single nucleotide polymorphism baseline for genetic stock identification of Chinook Salmon (*Oncorhynchus tshawytscha*) in the California current large marine ecosystem. Fish Bull.

[45] Edmands S (2007). Between a rock and a hard place: evaluating the relative risks of inbreeding and outbreeding for conservation and management. Mol Ecol.

[46] Frankham R, Ballou JD, Eldridge MDB, Lacy RC, Ralls K, Dudash MR (2011). Predicting the probability of outbreeding depression. Conserv Biol.

[47] Scott PA, Allison LJ, Field KJ, Averill-Murray RC, Bradley SH (2020). Individual heterozygosity predicts translocation success in threatened desert tortoises. Science.

[48] Hufford K, Mazer S (2003). Plant ecotypes: genetic differentiation in the age of ecological restoration. Trends Ecol Evol.

[49] Lenormand T (2002). Gene flow and the limits to natural selection. Trends Ecol Evol.

[50] Davis MM, Smyser TJ, Johnson SA, Duchamp J, Larkin JL, Swihart RK (2021). Reproductive success of captive-reared Allegheny Woodrats (*Neotoma magister*) released into genetically depauperate populations. Conserv Genet.

[51] Lotsander A, Hasselgren M, Larm M, Wallén J, Angerbjörn A, Norén K (2021). Low persistence of genetic rescue across generations in the arctic fox (*Vulpes lagopus*). J Hered.

[52] Gustafson KD, Vickers TW, Boyce WM, Ernest HB (2017). A single migrant enhances the genetic diversity of an inbred puma population. R Soc Open Sci.

[53] Vilà C, Sundqvist AK, Flagstad Ø, Seddon J, Björnerfeldt S, Kojola I (2003). Rescue of a severely bottlenecked wolf (*Canis lupus*) population by a single immigrant. Proc R Soc B.

[54] Hedrick PW, Peterson RO, Vucetich LM, Adams JR, Vucetich JA (2014). Genetic rescue in Isle Royale wolves: genetic analysis and the collapse of the population. Conserv Genet.

[55] Hedrick P, Robinson J, Peterson R, Vucetich J (2019). Genetics and extinction and the example of Isle Royale wolves. Anim Conserv.

[56] Robinson JA, Räikkönen J, Vucetich LM, Vucetich JA, Peterson RO, Lohmueller KE (2019). Genomic signatures of extensive inbreeding in Isle Royale wolves, a population on the threshold of extinction. Sci Adv.

[57] Stoffel MA, Humble E, Paijmans AJ, Acevedo-Whitehouse K, Chilvers BL, Dickerson B (2018). Demographic histories and genetic diversity across pinnipeds are shaped by human exploitation, ecology and life-history. Nat Commun.

[58] Jones C, Noble L, Jones J, Tegelstrom H, Triggs G, Berry R (1995). Differential male genetic success determines gene flow in an experimentally manipulated mouse population. Proc R Soc B.

[59] Ingvarsson PK, Whitlock MC (2000). Heterosis increases the effective migration rate. Proc R Soc B.

[60] Heinsohn R, Ebert D, Legge S, Peakall R (2007). Genetic evidence for cooperative polyandry in reverse dichromatic Eclectus parrots. Anim Behav.

[61] Saccheri IJ, Brakefield PM (2002). Rapid spread of immigrant genomes into inbred populations. Proc R Soc B.

[62] Allendorf FW, Hohenlohe P, Luikart G (2010). Genomics and the future of conservation genetics. Nat Rev Genetics..

[63] Kanine JM, Kierepka EM, Castleberry SB, Mengak MT, Nibbelink NP, Glenn TC (2018). Influence of landscape heterogeneity on the functional connectivity of Allegheny woodrats (*Neotoma magister*) in Virginia. Conserv Genet.

[64] Bolger A, Lohse M, Usadel B (2014). Trimmomatic: a flexible trimmer for Illumina sequence data. Bioinformatics.

[65] Simpson JT, Wong K, Jackman SD, Schein JE, Jones SJM, Birol I (2009). ABySS: a parallel assembler for short read sequence data. Genome Res.

[66] Simao FA, Waterhouse RM, Ioannidis P, Kriventseva EV, Zdobnov EM (2015). BUSCO: assessing genome assembly and annotation completeness with single-copy orthologs. Bioinformatics.

[67] Nishimura O, Hara Y, Kuraku S (2017). gVolante for standardizing completeness assessment of genome and transcriptome assemblies. Bioinformatics.

[68] Cantarel BL, Korf I, Robb SMC, Parra G, Ross E, Moore B (2008). MAKER: an easy-to-use annotation pipeline designed for emerging model organism genomes. Genome Res.

[69] Doyle JM, Katzner TE, Bloom PH, Ji Y, Wijayawardena BK, DeWoody JA (2014). The genome sequence of a widespread apex predator, the golden eagle (*Aquila chrysaetos*). PLoS ONE.

[70] Korf I (2004). Gene finding in novel genomes. BMC Bioinformatics.

[71] Li H, Durbin R (2009). Fast and accurate short read alignment with Burrows-Wheeler transform. Bioinformatics.

[72] van der Auwera GA, Carneiro MO, Hartl C, Poplin R, del Angel G, Levy-Moonshine A (2013). From fastQ data to high-confidence variant calls: the genome analysis toolkit best practices pipeline. Current Protoc Bioinf..

[73] DePristo MA, Banks E, Poplin R, Garimella K, Maguire JR, Hartl C (2011). A framework for variation discovery and genotyping using next-generation DNA sequencing data. Nat Genet.

[74] Quinlan AR, Hall IM (2010). BEDTools : a flexible suite of utilities for comparing genomic features. Bioinformatics.

[75] Cingolani P, Platts A, Wang L, Coon M, Nguyen T, Wang L (2012). A program for annotating and predicting the effects of single nucleotide polymorphisms, SnpEff: SNPs in the genome of *Drosophila melanogaster* strain w1118; iso-2, iso-3. Fly.

[76] Thorvaldsdóttir H, Robinson J, Mesirov J (2013). Integrative Genomics Viewer (IGV): high-performance genomics data visualization and exploration. Brief Bioinform.

[77] Caughley G (1977). Analysis of vertebrate populations.

[78] Johnson SA (2002). Reassessment of the Allegheny woodrat (*Neotoma magister*) in Indiana. Proc Indiana Acad Sci.

[79] Mengak M, Butchkoski C, Feller D, Johnson S, Peles J, Wright J (2008). Lessons from long-term monitoring of woodrat populations. The Allegheny Woodrat: ecology, conservation, and management of a declining species.

[80] Clayton D. snpStats: SnpMatrix and XSnpMatrix classes and methods. 2014.

[81] Kalinowski ST, Taper ML, Marshall TC (2007). Revising how the computer program CERVUS accommodates genotyping error increases success in paternity assignment. Mol Ecol.

[82] Pritchard JK, Stephens M, Donnelly P (2000). Inference of population structure using multilocus genotype data. Genetics.

[83] Falush D, Stephens M, Pritchard JK (2003). Inference of population structure using multilocus genotype data: linked loci and correlated allele frequencies. Genetics.

[84] Castleberry SB, King TL, Wood PB, Ford WM (2002). Microsatellite DNA analysis of population structure in Allegheny woodrats (*Neotoma magister*). J Mammal.

[85] Smyser TJ, Johnson SA, Page LK, Hudson CM, Rhodes OE (2013). Use of experimental translocations of allegheny woodrat to decipher causal agents of decline. Conserv Biol.

[86] Earl D, VonHoldt BM (2011). STRUCTURE HARVESTER: a website and program for visualizing STRUCTURE output and implementing the Evanno method. Conserv Genet Resour.

[87] Kopelman NM, Mayzel J, Jakobsson M, Rosenberg NA, Mayrose I (2015). CLUMPAK: A program for identifying clustering modes and packaging population structure inferences across K. Mol Ecol Resour.

[88] Peakall R, Smouse PE (2012). GenAlEx 6,5: genetic analysis in excel population genetic software for teaching and research–an update. Bioinformatics.

[89] DeWoody J, Avise JC (2000). Microsatellite variation in marine, freshwater and anadromous fishes compared with other animals. J Fish Biol.

[90] Eo S, Doyle J, DeWoody J (2011). Genetic diversity in birds is associated with body mass and habitat type. J Zool.

[91] Doyle JM, Hacking CC, Willoughby JR, Sundaram M, DeWoody JA (2015). Mammalian genetic diversity as a function of habitat, body size, trophic class, and conservation Status. J Mammal.

[92] Willoughby JR, Sundaram M, Wijayawardena BK, Kimble SJA, Ji Y, Fernandez NB (2015). The reduction of genetic diversity in threatened vertebrates and new recommendations regarding IUCN conservation rankings. Biol Conserv.

[93] Willoughby JR, Sundaram M, Wijayawardena BK, Lamb MC, Kimble SJA, Ji Y (2017). Biome and migratory behaviour significantly influence vertebrate genetic diversity. Biol J Lin Soc.

[94] Schunter C, Garza JC, Macpherson E, Pascual M (2014). SNP development from RNA-seq data in a nonmodel fish: how many individuals are needed for accurate allele frequency prediction?. Mol Ecol Resour.

[95] Baetscher DS, Hasselman DJ, Reid K, Palkovacs EP, Garza JC (2017). Discovery and characterization of single nucleotide polymorphisms in two anadromous alosine fishes of conservation concern. Ecol Evol.

[96] Reid K, Palkovacs EP, Hasselman DJ, Baetscher D, Kibele J, Gahagan B (2018). Comprehensive evaluation of genetic population structure for anadromous river herring with single nucleotide polymorphism data. Fish Res.

[97] Davis CD, Garza JC, Banks MA (2017). Identification of multiple genetically distinct populations of Chinook salmon (*Oncorhynchus tshawytscha*) in a small coastal watershed. Environ Biol Fishes.

[98] Ford MJ, Murdoch AR, Hughes MS, Seamons TR, Lahood S (2016). Broodstock history strongly influences natural spawning success in hatchery Steelhead (*Oncorhynchus mykiss*). PLoS ONE.

[99] Katzner TE, Nelson DM, Braham MA, Doyle JM, Fernandez NB, Duerr AE (2017). Golden Eagle fatalities and the continental-scale consequences of local wind-energy generation. Conserv Biol.

[100] Mathur S, Tomeček JM, Heniff A, Luna R, DeWoody JA (2019). Evidence of genetic erosion in a peripheral population of a North American game bird: the Montezuma quail (*Cyrtonyx montezumae*). Conserv Genet.

[101] Turley B, Reece K, Shen J, Ho J, Ximing L, Jan G (2019). Multiple drivers of interannual oyster settlement and recruitment in the lower Chesapeake Bay. Conserv Genet.

[102] Turley BD. Oyster reef connectivity inferred via population genetic analysis. 2015.

[103] Day GQ, Ng J, Oldt RF, Houghton PW, Smith DG, Kanthaswamy S (2018). DNA-based determination of ancestry in Cynomolgus Macaques (*Macaca fascicularis*). J Am Assoc Lab Anim Sci.

