# Supplementary material for: A novel SNP assay reveals increased genetic variability and abundance following translocations to a remnant Allegheny woodrat population
Source: BMC Ecol Evol. 2022 Nov 24;22:137. doi: 10.1186/s12862-022-02083-w (PMC9686018; doi:10.1186/s12862-022-02083-w)
Supplement: Supplementary file 2 — Additional file 2: Summary statistics associated with nuclear genome sequencing, assembly and annotation. [file 12862_2022_2083_MOESM2_ESM.docx]

*Supplementary File 2.docx: Supplementary File 2. Summary statistics associated with nuclear genome sequencing, assembly and annotation*

We generated 137.6 gigabases (Gb) of raw sequence data from *N. magister*, including 119.8 Gb from the paired-end (PE) library and 17.8 Gb from the mate-paired (MP) library (SI Table 1). Our draft nuclear genome assembly includes 60,789 scaffolds greater than 2,000 basepairs (bp) in length. These scaffolds had an N50 of 82 kilobases (kb) and the longest scaffold was 792 kb in length. We annotated 42,986 scaffolds greater than 10 kb (shorter scaffolds rarely produce high-quality gene annotations; C. Holt, personal communication). This process produced 21,151 gene annotations. The median gene length was 16,159 bp with median exon and intron lengths of 125 bp and 970 bp, respectively. The median number of exons was 6.

SI Table 1: Summary statistics for Allegheny woodrat (*Neotoma magister*) PE and MP libraries.

| Library | Raw data | | Following quality control | |
| --- | --- | --- | --- | --- |
|  | Total data (Gb) | Total reads | Total data (Gb) | Total reads |
| PE | 119.8 | 1,186,423,698 | 114.0 | 1,148,799,108 |
| MP | 17.8 | 176,221,284 | 10.5 | 120,411,652 |
