# Supplementary material for: A novel SNP assay reveals increased genetic variability and abundance following translocations to a remnant Allegheny woodrat population
Source: BMC Ecol Evol. 2022 Nov 24;22:137. doi: 10.1186/s12862-022-02083-w (PMC9686018; doi:10.1186/s12862-022-02083-w)
Supplement: Supplementary file 4 — Additional file 4: Relationship between probability of identity (PID), probability of identity between siblings (PIDsib) and the number of genotyped SNP or microsatellite loci. [file 12862_2022_2083_MOESM4_ESM.docx]

*Supplementary File 4.docx: Supplementary File 4. Relationship between probability of identity (P_ID_), probability of identity between siblings (PIDsib) and the number of genotyped SNP or microsatellite loci*

We conducted preliminary analyses to explore whether the SNP assay provides greater statistical power for individual identification than a commonly used panel of microsatellite markers. We genotyped 50 woodrats captured in 2017 and 2019 in Adams County, Ohio (OH) at 11 microsatellite markers consisting of Nma01, Nma02, Nma4, Nma5, Nma6, Nma8, Nma10, Nma11, Nma12, Nma14 and Nma15 (Castleberry et al. 2002; Smyser et al. 2012, 2013). This resulted in a P_ID_ (the likelihood that two randomly chosen individuals in a population will present seemingly identical genotypes; Paetkau and Strobeck 1994) of 4.0 x 10-5 and a probability of identity among siblings (PIDsib) of 9.8 x 10-3 (SI Figures 1 and 2). By contrast, 134 SNP loci generate values of 5.0 x 10-27 (P_ID_) and 3.1 x 10-14 (PIDsib). If the more conservative data set of 70 loci is used, the PID is 1.9 x 10-13 and the PIDsib is 3.1 x 10-7 across all 50 individuals. Furthermore, our results indicate that a much smaller panel of SNPs might be utilized in subsequent studies to achieve a P_ID_ <0.0001 (SI Figures 1 and 2). A P_ID_ < 0.0001 is considered low enough to distinguish between even closely related individuals in most natural populations (Waits et al. 2001; Thaden et al. 2020). To aid visualization of the relationship between P_ID_, PIDsib and number of loci, we plotted just a maximum number of 50 loci and a maximum probability of identity of 0.1000 (SI Figure 1) or 0.0010 (SI Figure 2).


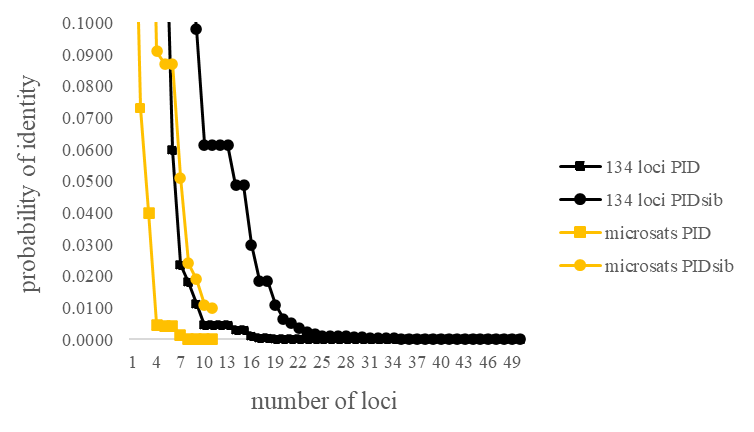


SI Figure 1: Relationship between probability of identity (P_ID_), probability of identity between siblings (PIDsib) and the number of genotyped SNP or microsatellite loci. Probability of identity decreases as the number of loci associated with each panel increases. To aid visualization, we show only a maximum number of 50 loci and a maximum probability of identity of 0.1000.


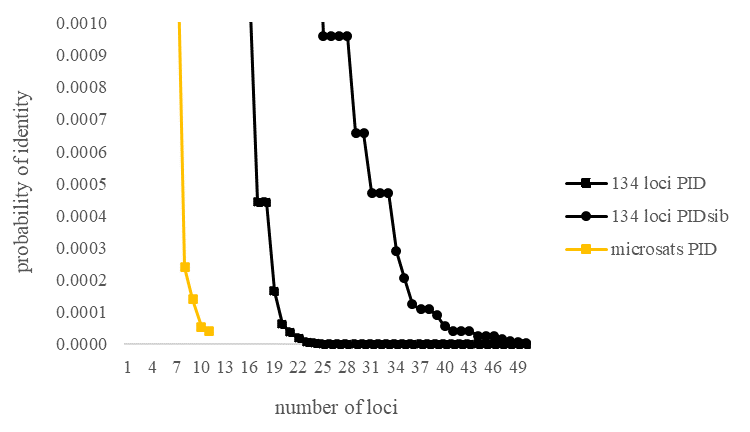


SI Figure 2: Relationship between probability of identity (P_ID_), probability of identity between siblings (PIDsib) and the number of genotyped SNP or microsatellite loci. Probability of identity decreases as the number of loci associated with each panel increases. To aid visualization, we show only a maximum number of 50 loci and a maximum probability of identity of 0.0010. The PIDsib associated with the microsatellite panel of 11 markers was greater than 0.0010 and is therefore not included here.

**Literature cited**

Castleberry SB, King TL, Wood PB, Ford WM (2002) Microsatellite DNA analysis of population structure in Allegheny woodrats (*Neotoma magister*). Journal of Mammalogy 83:1058–1070. https://doi.org/10.1644/1545-1542(2002)083<1058:MDAOPS>2.0.CO;2

Paetkau D, Strobeck C (1994) Microsatellite analysis of genetic variation in black bear populations. Mol Ecol 3:489–95

Smyser TJ, Duchamp JE, Johnson SA, et al (2012) Consequences of metapopulation collapse: Comparison of genetic attributes between two Allegheny woodrat metapopulations. Conservation Genetics 13:849–858. https://doi.org/10.1007/s10592-012-0334-1

Smyser TJ, Johnson SA, Page LK, et al (2013) Use of experimental translocations of Allegheny woodrat to decipher causal agents of decline. Conservation Biology 27:752–762. https://doi.org/10.1111/cobi.12064

Thaden A von, Nowak C, Tiesmeyer A, et al (2020) Applying genomic data in wildlife monitoring: Development guidelines for genotyping degraded samples with reduced single nucleotide polymorphism panels. Molecular Ecology Resources 20:662–680. https://doi.org/10.1111/1755-0998.13136

Waits LP, Luikart G, Taberlet P (2001) Estimating the probability of identity among genotypes in natural populations: Cautions and guidelines. Molecular Ecology 10:249–256. https://doi.org/10.1046/j.1365-294X.2001.01185.x
