# Supplementary material for: A novel SNP assay reveals increased genetic variability and abundance following translocations to a remnant Allegheny woodrat population
Source: BMC Ecol Evol. 2022 Nov 24;22:137. doi: 10.1186/s12862-022-02083-w (PMC9686018; doi:10.1186/s12862-022-02083-w)
Supplement: Supplementary file 5 — Additional file 5: Comparison of microsatellite- and SNP-based genetic variation. [file 12862_2022_2083_MOESM5_ESM.docx]

*Supplementary File 5.docx: Supplementary File 5. Comparison of microsatellite- and SNP-based genetic variation*

Fifty of our samples (collected in Ohio between 2017 and 2019) underwent microsatellite genotyping in addition to SNP genotyping to compare the genetic variation associated with microsatellite and SNP assays. Individuals were genotyped at 11 microsatellite loci consisting of Nma01, Nma02, Nma4, Nma5, Nma6, Nma8, Nma10, Nma11, Nma12, Nma14 and Nma15 (Castleberry et al. 2002; Smyser et al. 2012; Smyser et al. 2013). Individuals were heterozygous at 1-6 of the 11 microsatellite markers (H_O_ = 0.27 ± 0.06, H_E_ = 0.38 ± 0.08) and 24-44 of the 134 SNP markers. There was a significant, positive relationship between the number of heterozygous microsatellite loci and heterozygous SNP loci per individual (linear regression: r^2^ = 0.32, p < 0.0001; SI Figure 3). Microsatellite H_o_ and H_e_ were calculated with GenAlEx 6.501 (Peakall and Smouse 2012).


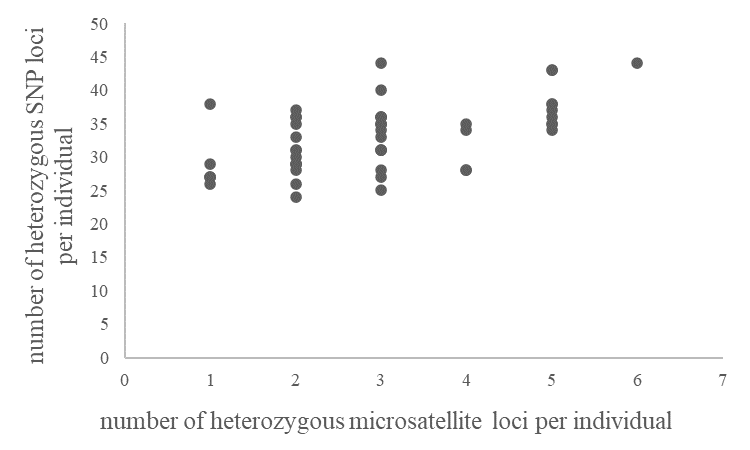


SI Figure 3: There was a significant, positive relationship between the number of heterozygous microsatellite loci and heterozygous SNP loci per individual (linear regression: r^2^ = 0.32, p < 0.0001).

**Literature cited**

Castleberry SB, King TL, Wood PB, Ford WM (2002) Microsatellite DNA analysis of population structure in Allegheny woodrats (Neotoma magister). J Mammal 83:1058–1070. doi: 10.1644/1545-1542(2002)083<1058:MDAOPS>2.0.CO;2

Peakall R, Smouse PE (2012) GenAlEx 6.5: genetic analysis in Excel. Population genetic software for teaching and research--an update. Bioinformatics 28:2537–9. doi: 10.1093/bioinformatics/bts460

Smyser TJ, Duchamp JE, Johnson SA, et al (2012) Consequences of metapopulation collapse: Comparison of genetic attributes between two Allegheny woodrat metapopulations. Conserv Genet 13:849–858. doi: 10.1007/s10592-012-0334-1

Smyser TJ, Johnson SA, Page LK, et al (2013) Use of experimental translocations of allegheny woodrat to decipher causal agents of decline. Conserv Biol 27:752–762. doi: 10.1111/cobi.12064
